# Ras Isoprenylation and pAkt Inhibition by Zoledronic Acid and Fluvastatin Enhances Paclitaxel Activity in T24 Bladder Cancer Cells

**DOI:** 10.3390/cancers3010662

**Published:** 2011-02-14

**Authors:** Shaojuan Li, Paul de Souza

**Affiliations:** 1 Cancer Pharmacology and Therapeutics Laboratory, St George Hospital, Kogarah, NSW, Australia; E-Mail: Shaojuan.Li@sesiahs.health.nsw.gov.au; 2 St George Hospital Clinical School, University of New South Wales, New South Wales, Australia

**Keywords:** synergy, taxanes, Ras, AKT, signaling

## Abstract

**Background:**

Bisphosphonates interfere with the mevalonate pathway and inhibit the prenylation of small GTP-binding proteins such as ras and rap. We hypothesized that zoledronic acid would synergistically inhibit T24 bladder cancer cell growth in combination with fluvastatin and paclitaxel.

**Methods:**

Increasing doses of fluvastatin, zoledronic acid, and paclitaxel were investigated as single agents and in combination, and synergistic interactions were evaluated by the Chou-Talalay method. Western blots were used to assess effects on signal transduction pathways.

**Results:**

Growth of T24 was significantly inhibited with IC_50_ values of 2.67 ± 0.61 μM for fluvastatin and 5.35 ± 1.35 μM for zoledronic acid after 72 hours treatment. Geranylgeranyl pyrophosphate and farnesyl pyrophosphate was able to block, in part, this inhibitory activity. The combinations of zoledronic acid and paclitaxel, zoledronic acid and fluvastatin, and fluvastatin and paclitaxel were all synergistic. Both fluvastatin and zoledronic acid inhibited Ras and Rap prenylation, and the phosphorylation of ERK1/2 and AKT. The degree of inhibition of phosphorylation of these key signaling transduction pathways appears to closely correlate with their synergistic interactions.

**Conclusions:**

Zoledronic acid enhances fluvastatin and paclitaxel activity against T24 in a synergistic manner and this is mediated largely by inhibition of both the Ras/Raf/MEK/ERK and PI3K/AKT signaling pathways via isoprenylation inhibition.

## 1. Introduction

Protein prenylation is the covalent addition of a 15-carbon farnesyl or a 20-carbon geranylgeranyl isoprenoid onto acceptor proteins [1]. The enzymes farnesyl transferase and geranylgeranyl transferase catalyse the transfer and subsequent binding of farnesyl and geranylgeranyl isoprenoid moieties from farnesylpyrophosphate (FPP) and geranylgeranylpyrophosphate (GGPP) to the C-terminus of a target protein in the mevalonate synthesis pathway [1]. Prenylation is essential for membrane attachment [2,3] and the subsequent participation of prenylated proteins in diverse signaling pathways regulating cell growth and survival [4,5]. Proteins that require geranylgeranylation or farneslation for their function include small GTP-binding proteins such as Rho family members rha, rab, rac and cdc-42 (geranylgeranylation), and Ras family members Rap (geranylgeranylation) and ras (farnesylation) [1,5,6]. Ras, a 21 kD GTP binding protein is a key intersecting molecule involved in several key signaling pathways including Raf/MEK/ERK and PI3K/AKT [7,8]. Mutational activation transforms Ras into an oncogenic form that continually sends positive signals favoring cell proliferation [9]. It is estimated that approximately 30% of all human cancers carry a variety of Ras mutations; molecular epidemiologic studies show that up to 80% of bladder transitional cell carcinoma carry mutational activated H-ras [10-12]. Oncogene H-ras is overexpressed in the T24 bladder cancer cell line [13] and is thus an attractive model to study the signal transduction inhibitory effects of zoledronic acid.

Bisphosphonates are class of drugs widely used for treatment of osteoporosis and bone disorders including tumor-associated bone disease. Recently, it has been shown that nitrogen-containing bisphosphonates such as zoledronic acid inhibit farnesyldiphosphate synthase [14]. We hypothesized that zoledronic acid would inhibit the growth of the T24 bladder cancer cell line, and that this activity would be enhanced by fluvastatin and with paclitaxel. Further, given the mechanism of action of zoledronic acid on farnesylation and geranylgeranylation, we hypothesized that any synergistic interaction with fluvastatin and paclitaxel might be mediated via isoprenylation inhibition.

## 2. Results and Discussion

### 2.1. Growth Inhibition of T24 Cells by Fluvastatin and Zoledronic Acid

**Figure 1.**
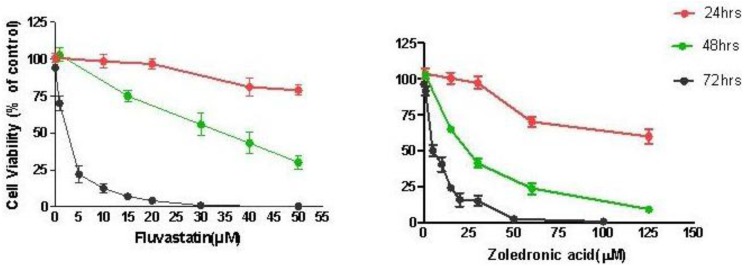
*In vitro* cytotoxicity of Fluvastatin and Zoledronic acid. T24 cells were exposed to a range of concentrations of fluvastatin (0.1 to 50 μM) and zoledronic acid (0.1 to 125 μM) for 24 to 72 hours as indicated, control cells treated identically except without adding drugs, the percentage of alive cells were detected by SRB method. The cytotocxicity of both drugs appeared dose and time dependant, T24 cells are slightly more sensitive to fluvastatin than to zoledronic acid, with an IC_50_ of 2.67 ± 0.61 μM for fluvastatin and 5.35 ± 1.35 μM for zoledronic acid at 72 hours treatment. Data represent the mean ± SD from three separate experiments.

Treatment of T24 cells with increasing concentrations of fluvastatin (0.1 to 100 μM) or zoledronic acid (0.1 to 100 μM) for 24–72 hours caused dose and time-dependent cell growth inhibition. T24 cells were slightly more sensitive to fluvastatin (IC_50_ 2.67 ± 0.61 μM at 72 h) than to zoledronic acid (IC_50_ 5.35 ± 1.35 μM at 72 h) (Figure 1).

### 2.2. Geranylgeranyl Pyrophosphate and Farnesyl Pyrophosphate Rescue Growth Inhibition of T24 Cells Induced by Fluvastatin and Zoledronic Acid

**Figure 2.**
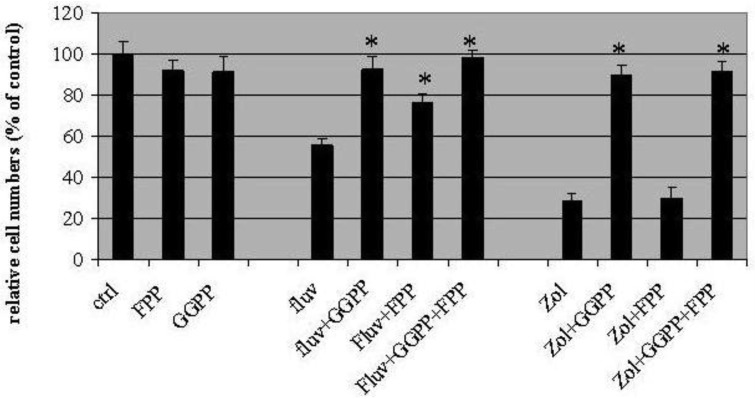
Reversal of cell growth inhibition induced by fluvastatin (Fluv) and zoledronic acid (Zol) with geranylgeranyl pyrophosphate (GGPP) and farnesyl pyrophosphate (FPP). Control cells were maintained in growth medium and represent 100% cell growth. GGPP significantly rescues cell death from both drugs (P < 0.01, n = 3), but FPP only blocks cell growth inhibition induced by fluvastatin (P < 0.01, n = 3). There is no significant difference between zoledronic acid alone and zoledronic acid + FPP (P = 0.388, n = 3). Data represent the mean ± SD from three separate experiments.

In these experiments, fluvastatin (3μM) and zoledronic acid (10μM) inhibited cell proliferation by 44 ± 6.2%, and 71 ± 2%, respectively (Figure 2). Geranylgeranyl pyrophosphate (GGPP, 10 μM) appeared to completely block T24 growth inhibition induced by the two drugs, with the proportion of surviving cells similar to control values (91 ± 8% for fluvastatin and 89 ± 6% for zoledronic acid). On the other hand, farneysl pyrophosphate (FPP) could only partially rescue the growth inhibition induced by fluvastatin (76.3 ± 4% of control) while leaving cytotoxicity of zoledronic acid unaffected (30 ± 5% of control). Growth inhibition by both fluvastatin and zoledronic acid could be rescued completely by adding GGPP and FPP in combination (98 ± 4% control for fluvastatin and 91 ± 5% control for zoledronic acid).

### 2.3. Fluvastatin and Zoledronic Acid Combined with PaclitaxeI Results in Synergistic Growth Effects on T24 Cells

At lower concentrations of fluvastatin (0.5–1.5 μM), zoledronic acid (0.5–3 μM), and paclitaxel (below 2 nM) additive growth inhibition was achieved (CI = 0.9–1.1), whereas 5–10 μM of zoledronic acid and 1.5–3 μM fluvastatin combined with 4–8 nM of paclitaxel produced greater synergistic effects (combination indexes ranging from 0.3 to 0.6) (summary combination indexes only in Table 1).

### 2.4. Fluvastatin and Zoledronic Acid Inhibit Protein Prenylation of RAS and RAP

**Figure 3.**
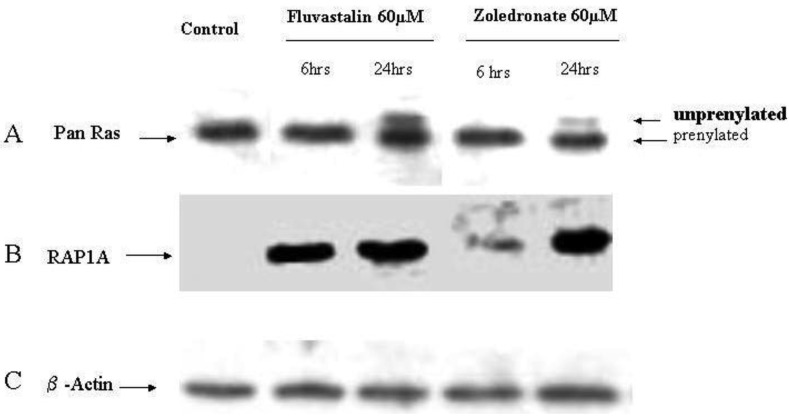
Inhibition of Ras and Rap prenylation by fluvastatin and zoledronic acid on T24 cells. T24 cells were grown in growth medium for 24 hours before harvest (control), or in growth medium containing the indicated drug for 6 to 24 hours. (**A**) anti-Ras antibody (farnesylation marker) can bind to both unprenylated and the prenylated ras. The unfarnesylated form migrates more slowly than the farnesylated form as indicated on the right side of the panel. Both drugs inhibit Ras farnesylation at 24h treatment but not 6h. (**B**) anti-Rap1A (geranylgeranylation marker) can only bind to the unprenylated Rap, and results show that fluvastatin inhibits Rap geranygeranylation at both 6 and 24 hours treatment, while zoledronic acid shows greater inhibition at 24h treatment and less inhibition at 6h. (**C**) β-actin as loading control.

In order to investigate the molecular mechanisms of fluvastatin and zoledronic acid induced cell growth inhibition, the effects of these two drugs on protein prenylation of Ras and Rap1 were analyzed by Western blot. Results show that both drugs inhibit Ras farnesylation after 24 hours treatment but not six hours (Figure 3). Fluvastatin inhibits Rap geranygeranylation at both six and 24 hours treatment, while zoledronic acid show greater inhibition at 24 hours treatment and less inhibition at six hours (Figure 3).

### 2.5. Reduced phosphorylation of ERK and AKT by fluvastatin and zoledronic acid

**Figure 4.**
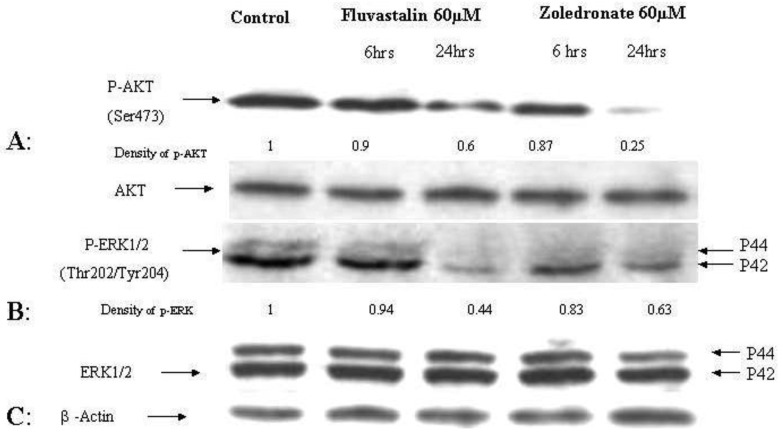
Inhibition of phosphorylation of ERK and AKT by fluvastatin and zoledronic acid. p-AKT (A) and p-ERK1/2 (B) level in T24 cells treated by fluvastatin and zoledronic acid *versus* control (medium only). β-actin is used as a loading control (C). Semiquantitative analysis of band signal intensity using densitometry is also shown.

The effects of the fluvastatin and zoledronic acid on signal transduction pathways of Ras/Raf/MEK/ERK and PI3K /AKT were investigated under the same conditions as above. Drug treatment showed a time-dependent decrease in phosphorylation of AKT and ERK1/2 (Figure 4).

**Figure 5.**
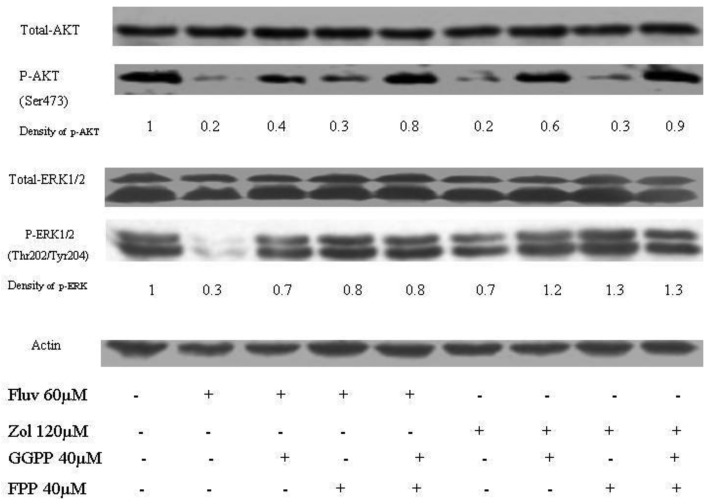
ERK1/2 and AKT pathway inhibition following treatment with fluvastatin and zoledronic acid, with or without rescue by GGPP and FPP. Western blotting results show that GGPP partially restores both phosphorylation of AKT and ERK1/2, while FPP restores phosphorylation of ERK1/2 and has less effect on AKT.

Using densitometry of protein expression, we estimate that zoledronic acid can inhibit the phosphorylation of AKT by up to 75%, and ERK1/2 by up to 36% at 24h. Further, the combination of fluvastatin and zoledronic acid inhibits pAKT and pERK to a greater degree than either drug alone, so that only approximately 20–30% of phosphorylation activity remains, as assessed by densitometry (Figure 5).

**Figure 6.**
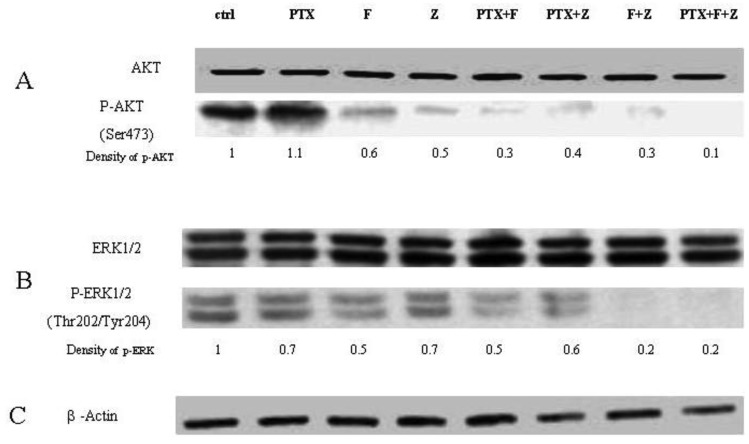
Inhibition of phosphorylation of AKT and ERK1/2 by paclitaxel (PTX), fluvastatin (F), zoledronic acid (Z) and their combinations. Data show that all combinations result in reduced phosphorylation of AKT (A) and ERK1/2 (B); the most effective combination appears to be paclitaxel + fluvastatin + zoledronic acid. β-actin is used as a loading control (C).

The Western blot confirms that phosphorylation of AKT is restored when GGPP is added back to either fluvastatin or zoledronic acid treatment, unlike phosporylation of ERK. When paclitaxel is added to the combination of fluvastatin and zoledronic acid, phosphorylation of AKT and ERK is almost completely abolished (final lane, Figure 6). The degree of inhibition observed with the different combinations seems to reflect very accurately the combination indexes noted by the Chou-Talalay method, suggesting that the mechanism for synergy could be explained in large part, to the perturbation of signal transduction inhibition.

### 2.6. Discussion

Whilst the observation that non-classical agents may have antiproliferative activity against cancer cells is not new, we found that both fluvastatin and zoledronic acid inhibited prenylation of Rap1 and Ras in the T24 bladder cancer cell line. Geranylgeranyl pyrophosphate and to a lesser extent, farnesyl pyrophosphate, are able to rescue cells from growth inhibition from fluvastatin. Geranylgeranyl pyrophosphate, but not farnesyl pyrophosphate, is able to do the same for zoledronic acid, suggesting that zoledronic acid preferentially inhibits geranylgeranylation. These data suggest that inhibition of geranylgeranylation and farnyesylation of GTP are important mechanisms of the anti-cancer effects of these two drugs.

Inhibition of the mevalonate pathway by fluvastatin and zoledronic acid leads to diminished synthesis of both FPP and GGPP. Our results demonstrate that GGPP was more effective at rescuing T24 cell growth than FPP. Moreover, the requirement of both FPP and GGPP to rescue zoledronic acid growth inhibition implies that these may be separate targets, and that inhibition of HMG Co-A reductase does not automatically cause the same cellular signaling effects. These data are supported by the observation in the MCF-7 breast cancer cell line, where 50 μM GGPP inhibited apoptosis similar to the control levels, while 50 μM FPP led to a partial inhibition of apoptosis [17]. Others have also noted that GGPP but not FPP can rescue nitrogen containing bisphosphonate (NBPs) induced cell death [18]. The reasons for the differences found in various cell lines are unclear, but it has been reported that inhibition of FPP synthase by many NBPs *in vitro* results in impaired FPP, and consequently GGPP production [14]. Others have also shown that NBPs have the ability to inhibit geranylgeranyl diphosphate synthase with an IC50 value of 97 μM [19] compared to 3 nM against FPP [14]. Our data are consistent with the notion that GGPP is more efficient than FPP for rescuing cell death induced by fluvastatin, and only GGPP but not FPP can rescue zoledronic acid induced cell death.

Statins and bisphosphonates inhibit critical enzymes [14,20,21] in the mevalonate pathway, resulting in inhibition of protein isoprenylation. The consequences of this inhibition are the disruption of the important signal transduction pathways that regulate proliferation and invasion. Since Ras, a 21kd protein, requires isoprenylation to activate downstream proliferative cascades of ERK and AKT [7], it is perhaps not surprising to find that phosphorylation of ERK does indeed occur with the use of fluvastatin and zoledronic acid in this cell line. Less expected was the demonstration that pAKT was also inhibited, as this is a key pathway for the intersection of multiple signal transduction pathways that cooperate to promote cell survival and proliferation [22,23], and is therefore likely to have multiple levels of redundancy. To our knowledge, the direct inhibition of AKT activity by zoledronic acid and fluvastatin has not been previously shown. Since H-ras has been found to be a potent activator of PI3K [7], we reasoned that Ras overexpression in this cell line might result in co-activation of PI3K/AKT pathway, so that blocking Ras function might explain inhibition of AKT function as well. Our Western blot data would suggest that GGPP and FPP have the ability to partially restore the phosphorylation of AKT (Figure 5), which is also a novel finding. This implies that restoring Ras function, mediated through rescue of isoprenylation inhibition, also results in partial restoration AKT function, providing strong evidence that Ras is acting upstream of AKT in this cell line. Further, there appears to be differential effects of GGPP and FPP on pAKT, depending on the agent used to inhibit Ras isoprenylation. We would be interested in the effect of fluvastatin and zoledronic acid on other Ras superfamily members that are farnesylated or geranylgeranylated (e.g., Ras-like, or RalA and RalB GTPases), since the relevance of Ral GTPases to bladder cancer has been previously reported [29,30].

Paclitaxel is a microtubule-targeted anticancer agent that binds to ®-tubulin and inducing mitotic arrest and apoptosis [24], and has single agent activity in patients with bladder cancer. It has a broad spectrum of antitumor activity, and has been reported to act synergistically in combination with many other drugs though the mechanisms of how these drugs sensitize each other have not been well investigated. One mechanism of paclitaxel resistance in cancer cells is due to the expression of the multidrug resistant protein MDR-1. Constant activation of PI3K/AKT can lead to MDR-1 over expression [25], so inhibition of signaling pathways including PI3K/AKT and MAPK could possibly re-sensitise cells to paclitaxel [26]. In this work, the combination of fluvastatin, zoledronic acid and paclitaxel generates synergistic anti-cancer effects on T24 *in vitro*, regardless of the particular two or three drug combination. Since we have shown that these drugs inhibit elements of both the Ras and AKT pathway, another potential mechanism for their synergistic action is the targeting of these key pathways. Indeed, the degree of abolition of both ERK1/2 and pAKT is striking when all three drugs are used in combination (Figure 6), raising the hypothesis that paclitaxel may have independent effects on these signal transduction pathways. Since paclitaxel has been shown to inhibit isoprenylation as well [27], it is plausible that the cause for synergistic activity for all three drugs in this cell line is the potent inhibition of Ras and AKT pathways. This suggests that a multiple target approach could potentially be a useful approach for the treatment of bladder cancer in the clinical setting. Further, as two of the drugs (fluvastatin, zoledronic acid) used in this combination are already used widely clinically and have minimal side effects, the combination with paclitaxel is very appealing and could provide a high benefit:risk ratio for clinical use. In our view, Phase I trials of this combination are warranted.

## 3. Experimental

### 3.1. Cell Culture and Reagents

Human bladder cancer cell line T24 cells were grown in RPMI medium (Sigma), supplemented with 10% of fetal calf serum (GIBCOBRL), 100 U/mL of penicillin and 100 μg/mL of streptomycin (GIBCOBRL), at a constant temperature of 37 °C in a humidified atmosphere with 5% CO_2_. Zoledronic acid (supplied as the hydrated disodium salt) was dissolved in ddH_2_O and fluvastatin was dissolved in DMSO; 100 mM of stock solutions were stored in −20 °C, and were diluted in growth medium before use. Both zoledronic acid and fluvastatin were kindly provided by Novartis Pharmaceuticals Australia. Paclitaxel (Sigma) was dissolved in DMSO and stored in 4 °C as a stock solution. Geranylgeranyl pyrophosphate and Farnesyl pyrophosphate were both purchased from Sigma and stored in −20 °C; Anti-RAP1A antibody, anti-® actin and horseradish peroxidase (HRP) -linked anti-goat antibody were purchased from Santa Cruz Biotechnology (U.S.). Anti-RAP1A antibody can bind to the nongeranylgeranylated form RAP1 [28]. Anti-phospho-AKT, anti-phospho-ERK1/2, anti-Pan Ras, HRP-linked anti-rabbit and anti-mouse secondary antibodies were purchased from Cell Signaling Tech (U.S.).

### 3.2. Growth Inhibition Assay

Cell growth inhibition was determined by the sulforhodamine B colorimetric assay (SRB) [15]. Briefly, T24 cells were seeded in 96-well plates with each well containing 1,000 cells. Drugs at different concentrations and different combinations were added after 24hrs and cells were incubated for a further 24 to 72 hours. Cells were then fixed with 10% of trichloroacetic acid at 4 °C for 50 minutes and washed with water for three times. Attached cells were then stained with 0.4% SRB (in 1% acetic acid) for 15 minutes at room temperature, then washed 3 times with 1% acetic acid, and when the plates were dried, 100 μL of 10 mM Tris base (pH 10.5) was added to each well, and the absorbance was measured at a wavelength of 570 nM in a microplate reader. Results are expressed as % control (medium alone) with standard errors of the mean (SEM), unless specified.

### 3.3. Rescue experiments

T24 cells were seeded in 96-well plates at 1000/well for 24 hours, then treated with the drugs (3 μM fluvastatin or 10 μM zoledronic acid) alone. Farneysl pyrophosphate and geranylgeranyl pyrophosphate (10 μM each) were then added within 60 minutes, and growth inhibition was assessed by SRB assay as above.

### 3.4. Synergy Assay for Drug Combination

Drug combination effects were analyzed using “CalcuSyn Windows Software for Dose Effect Analysis” developed by Chou and Talalay [16]. Briefly, the combination Index (CI) was used to determine drug interactions:

$$\text{CI}={\left(\text{D}\right)}_{1}/{\left({\text{D}}_{\text{X}}\right)}_{1}+{\left(\text{D}\right)}_{2}/{\left({\text{D}}_{\text{X}}\right)}_{2}+{\left(\text{D}\right)}_{1}{\left(\text{D}\right)}_{2}/{\left({\text{D}}_{\text{X}}\right)}_{1}{\left({\text{D}}_{\text{X}}\right)}_{2}$$

Where (D_X_)_1_ is the dose of drug-1 required to produce x percent effect alone and (D)_1_ is the dose of drug-1 required to produce the same x percent effect in combination with drug-2; similarly, (D_X_)_2_ is the dose of drug-2 required to produce x percent effect alone and (D)_2_ is the dose of drug-2 required to produce the same x percent effect in combination with drug-1. CI < 1, =1, and >1 indicates synergism, additive effect, and antagonism, respectively.

### 3.5. Western Blot Analysis

Cells were allowed to reach 70% confluence in RPMI containing 10% of FCS for 24 hours, before drugs were added in serum-free medium for another 24 hours. Cells were then washed with cold PBS buffer and lysed in buffer containing 50 mM Tris-HCI (pH7.5), 150 mM NaCI (150 mM), EGTA (1 mM), EDTA (2 mM), 2.5%(V/V) glycerol, NaF (10 mM), 0.2%(V/V) Triton X-100, 0.3% (V/V) NP40, aprotinin (5 μg/mL), Leupeptin (10 μg/mL), pepstatin (10 μg/mL), activated NaVO_3_ (2 mM) and PMSF (1 mM). Lysates were sonicated in an ice water bath for 20 seconds; the supernatant containing protein was separated from the insoluble material by centrifugation at 10,000rpm for 10 minutes at 4 °C. The total protein in each sample was then quantified using Bicinchoninic Acid Kit (Sigma) by following the protocol described. Samples containing equal amounts of protein were mixed with loading buffer and boiled for 5 minutes. Proteins were then separated by SDS-PAGE, and the proteins on the gels were transferred to PVDF-Membrane (Amersham Biosciences) and incubated with different antibodies. After incubation with HRP-conjugated secondary antibodies, the primary antibody binding was visualized with Western Lightning™ Chemiluminescence (PerkinElmer LAS, Inc.). Density of the bands were quantified by scanning with Gel Doc 200 (Bio-Rad) and normalized by β-actin as loading control (density of the sample /density of β-actin); P-AKT and P-ERK expression levels were further normalized by density of the band of the non-drug treated control. All Western blots were repeated at least once, and similar results were confirmed.

### 3.6. Statistical Analysis

Each experiment was performed at least three times, and all values are represented as the means ± SD of triplicates. Student's t-test was used to determine the statistical significance of the results. Values of p < 0.05 were considered statistically significant.

## 4. Conclusions

In conclusion, our paper demonstrates a number of novel observations of the mechanism of the antitumor activity of zoledronic acid on T24 cells. We show that zoledronic acid inhibits isoprenylation, and that this can be rescued by GGPP, but not by FPP. Further, isoprenylation inhibition inhibits the function of Ras, and this results in reduced phosphorylation of downstream targets such as ERK and AKT. The antiproliferative and anti-Ras function of zoledronic acid can be improved by the addition of fluvastatin and paclitaxel in a synergistic manner. The mechanism of this synergy appears to be largely due to drug effects on two major signaling transduction pathways, Ras, and AKT.

## Figures and Tables

**Table 1.** CI values for the drugs combinations at 50, 70 and 90% growth inhibition of T24 cells. CI<0.9 represents synergy, CI values between 0.9 and 1.1 indicate additive activity, and CI>1.1 represents antagonism.

**Drugs combinations**	**CI value ±SD**		

	**50%**	**70%**	**90%**
Zol + PTX	0.981 ± 0.05	0.726 ± 0.02	0.472 ± 0.09
Zol + Fluv	0.922 ± 0.12	0.856 ± 0.11	0.422 ± 0.08
Fluv + PTX	0.878 ± 0.03	0.811 ± 0.12	0.742 ± 0.03
Zol + Fluv + PTX	0.919 ± 0.11	0.774 ± 0.11	0.429 ± 0.06
